# The Effectiveness, Tolerability, and Safety of Different 1-Day Bowel Preparation Regimens for Pediatric Colonoscopy

**DOI:** 10.1155/2019/3230654

**Published:** 2019-11-03

**Authors:** Anna Szaflarska-Popławska, Dominika Tunowska, Ola Sobieska-Poszwa, Anna Gorecka, Aneta Krogulska

**Affiliations:** ^1^Department of Pediatric Endoscopy and Gastrointestinal Function Testing, Ludwik Rydygier Collegium Medicum in Bydgoszcz, Nicolaus Copernicus University in Torun, Poland; ^2^Department of Pediatrics, Allergology and Gastroenterology, Ludwik Rydygier Collegium Medicum in Bydgoszcz, Nicolaus Copernicus University in Torun, Poland

## Abstract

**Background:**

Currently, there is no generally accepted universal protocol for bowel preparation before colonoscopy in children.

**Aim:**

The aim of the study was to compare three different 1-day bowel preparation methods for a pediatric elective colonoscopy in terms of their efficacy, safety, and patient-reported tolerability. *Material and Methods*. The study was randomized, prospective, and investigator-blinded. All children aged 10 to 18 years consecutively referred to the tertiary pediatric gastroenterology unit were enrolled. The participants were randomized to receive polyethylene glycol 3350 combined with bisacodyl (PEG-bisacodyl group), or polyethylene glycol 4000 with electrolytes (PEG-ELS group), or sodium picosulphate plus magnesium oxide plus citric acid (NaPico+MgCit group). Bowel preparation was assessed according to the Boston Bowel Preparation Scale (BBPS). For patient tolerability and acceptability, questionnaires were obtained.

**Results:**

One hundred twenty-three children were allocated to three age- and sex-matched groups. All of the patients completed colonoscopies with visualization of the cecum. There was no difference among the groups for the mean BBPS score. A total of 73 patients (59.3%) experienced minor adverse events. No serious adverse events occurred in any group. Nausea was the only symptom more frequent in the PEG-ELS group compared to the NaPico+MgCit group (*p* = 0.04), and apathy was the only symptom more frequent in PEG-bisacodyl than in the NaPico+MgCit group (*p* = 0.04). All of the patients were able to complete 75% or more of the study protocol, and 85.4% were able to complete the full regimen. The acceptability was the highest in the NaPico+MgCit group with respect to the patient's grade for palatability, low volume of the solution, and willingness to repeat the same protocol.

**Conclusion:**

All bowel cleansing methods show similar efficacy. However, because of the higher tolerability and acceptability profile, the NaPico+MgCit-based regimen appears to be the most proper for colonoscopy preparation in children.

## 1. Introduction

Adequate bowel cleansing is crucial for various gastrointestinal diseases both in adults and children. Insufficient colonic cleanliness may result in missed diagnoses, prolonged procedure time, potential higher risk of colonoscopy-related complications, unsuccessful terminal ileum visualization, and increased financial burden from reexamination. The perfect preparation for colonoscopy should be low volume, short lasting, well tolerated, and efficient with no influence on the macroscopic picture of large bowel mucosa [1].

Bowel preparation regimens for pediatric colonoscopy vary greatly in terms of medication type and dosage, preparation length, and dietary restrictions among different institutions worldwide. There is no one standardized bowel cleansing schedule before colonoscopy in children. A recently published systematic review and meta-analysis on bowel preparation for elective procedures in children called for new randomized trials assessing efficacy, safety, and tolerability of bowel preparation in the pediatric population [2].

Bowel cleansing for pediatric colonoscopy procedures began in Poland with a clear liquid diet combined with laxatives such as senna and/or cleansing enemas in 2- to 4-day protocols. Then, cleansing protocol based on high-dose polyethylene glycol with electrolytes (PEG-ELS) for 2 days was implemented. However, the poor palatability, large preparation volumes, poor compliance with prolonged regimens, and strict dietary requirements were the disadvantages of these methods. Therefore, the new regimens based on low-volume polyethylene glycol and new agents having both osmotic and stimulant effects such as sodium picosulphate/magnesium citrate have been investigated.

Only a limited number of studies have evaluated the efficacy and safety of polyethylene glycol (PEG) 3350 powder in a 1-day bowel preparation regimen for pediatric colonoscopy [3–12]. Little is also known about the effectiveness and tolerability of sodium picosulphate/magnesium citrate before colonoscopy in children [9, 12–14].

### 1.1. Aim

The aim of the study was to evaluate bowel cleansing efficacy, safety, and patient-reported tolerance of three different 1-day bowel preparation regimens for the pediatric elective colonoscopy.

## 2. Materials and Methods

The study was designed as a randomized prospective trial and conducted at the university hospital between January 1, 2017, and November 30, 2018. The study was approved by the regional ethical committee of Ludwik Rydygier Collegium Medicum in Bydgoszcz, Poland (KB123/2017). Written informed consent was obtained from both parents and children ≥ 16 years or parents of children < 16 years of age at the time of enrollment.

Children aged ≥ 10 years who were consecutively referred to the Department of Pediatrics, Allergology and Gastroenterology of Jurasz University Hospital in Bydgoszcz, Poland, for elective colonoscopy were recruited. The participants were randomly assigned by an independent person to one of the three groups. Children younger than 10 years of age, with swallowing disorders, known allergy to one of the tested preparations, renal disease, or requiring an emergent colonoscopy were excluded.

The first group (PEG-bisacodyl group) received orally PEG-3350 (Dicopeg, Vitis Pharma) at a weight-adjusted dose (<50 kg: 4 g/kg body weight and ≥50 kg: 238 g) mixed in an isotonic, electrolyte-containing liquid used routinely for intravenous administration (<50 kg: 55 mL/kg body weight and ≥50 kg: 2 L) combined with bisacodyl (<50 kg: 5 mg and ≥50 kg: 10 mg). This protocol was recommended as a first-choice regimen for pediatric bowel preparation by the North American Society for Pediatric Gastroenterology, Hepatology and Nutrition [1].

The second group (PEG-ELS group) received orally PEG-ELS (Fortrans, Ipsen Pharma) at a weight-adjusted dose (100 mL/kg body weight to a maximum of 4 L) combined with simethicone (80 mg/1000 mL solution). Fortrans contains PEG 4000 with electrolytes: potassium chloride, sodium chloride, sodium bicarbonate, and sodium sulphate anhydrous.

The third group (NaPico+MgCit group) received 2 oral doses of sodium picosulphate plus magnesium oxide and citric acid, each (CitraFleet, Takeda) diluted in 150 mL of water. Intake of at least 40-50 mL/kg body weight to a maximum of 2 L of clear fluids was recommended. Every sachet of CitraFleet (15.08 g) contains sodium picosulphate, magnesium oxide, and citric acid anhydrous.

The patients were on a clear liquid diet the day before the colonoscopy until instructed not to take anything by mouth from 3:00 in the morning ranging 5 to 9 hours before the procedure based on anesthesia recommendations. The patients were instructed to start drinking preprocedure laxative at one o'clock the day before and complete the preparation in 6 hours. No additional rectal enemas or suppositories were provided.

The detailed verbal face-to-face bowel preparation instructions were communicated, and the questionnaire was provided to the parent and/or patient by the physician in charge. The participants were instructed to inform the doctor on duty when unable to complete more than 75% of the regimen in 6 hours. Data collected included demographic information, amount of bowel preparation completed, the degree of difficulty completing the prescribed bowel preparation, adverse symptoms developed during bowel preparation ingestion, and future willingness to use the same preparation.

The colonoscopy was carried out by two experienced pediatric endoscopists with more than 10 years' experience using i.v. anesthesia. The endoscopists were blinded to the preparation given. All of the participants were on a glucose drip under anesthesia. The effectiveness of the bowel cleansing was assessed according to the Boston Bowel Preparation Scale (BBPS). It is a 10-point (0-9 points) validated scoring system, based on a rating of 0 to 3 in 3 sections of the colon (right colon, transverse colon with hepatic and splenic flexures, and left colon), where 0 means unprepared colon due to solid stool, 1 means portion of mucosa not seen, 2 means minor amount of residual staining, and 3 means entire mucosa seen with minimal stool or fluid. The BBPS is graded from 0 to 9 points. A total BBPS score ≥ 6 and/or subscore ≥ 2 in each colon section was defined as adequate bowel preparation [15].

For all statistical analyses, software package SPSS (IBM® SPSS® statistics) were used. For assessing the relationship between categorical variables, contingency tables and the Chi-squared test were used. For analyzing the significance of differences in groups, the Mann-Whitney *U*-test (when comparing two groups) and the Kruskal-Wallis test (when comparing more than two groups) were used. BBPS scores were additionally compared after categorization into a binary variable, at least 6 and >6. A *p* value of <0.05 was considered statistically significant.

## 3. Results

In total, 123 children were enrolled in the study. Of those, 41 were allocated to the PEG-bisacodyl group, 43 to the PEG-ELS group, and 39 to the NaPico+MgCit group. There were 62 boys (50.4%) and 61 girls (49.6%) with a mean age of 14.1 (range 10-17) and mean weight of 53.2 kg. The indications for elective colonoscopy included suspected or known inflammatory bowel disease in 103 (83.7%) patients and others in 20 (16.3%) patients, e.g., rectal bleeding and/or abdominal pain in 14, inflammation other than inflammatory bowel disease in 2, anemia of unknown etiology in 2, and suspicion of familiar adenomatous polyposis in 2 patients. Constipation before bowel cleansing was reported by 4 patients. There were no differences between the 3 study groups regarding age, sex, mean weight, and reason for colonoscopy. The patient demographics and reason for colonoscopy are presented in Table 1.

### 3.1. Bowel Cleansing Effect

All of the patients had completed colonoscopies with the cecum visualized in 100%. An adequate bowel cleansing (BBPS ≥ 6 points) was observed in 36/41 (87.8%) of PEG-bisacodyl patients, 38/43 (88.4%) of PEG-ELS patients, and 34/39 (87.2%) of NaPico+MgCit patients, and the differences were not statistically significant (*p* = 0.987). The mean total BBPS score was 7.4 ± 1.6 in PEG-bisacodyl group, 7.6 ± 1.4 in PEG-ELS group, and 7.5 ± 1.5 in NaPico+MgCit group. The differences for the mean BBPS score among the 3 groups were not statistically significant (PEG-bisacodyl vs. PEG-ELS: *p* = 0.6, PEG-bisacodyl vs. NaPico+MgCit: *p* = 0.6, and PEG-ELS vs. NaPico+MgCit: *p* = 0.9). Only 3 of the patients (one from each study group) received a BBPS ≤ 4 and were considered for a repeated colonoscopy because of improper preparation for the procedure.

### 3.2. Tolerability

A total of 73 patients of 123 (59.3%) experienced mild adverse events, including abdominal pain, nausea, vomiting, flatulence, perianal discomfort/pain, sleep disturbance, dizziness, and apathy. No serious adverse events occurred in any group. There were no significant differences found in the prevalence of the adverse events between the 3 study groups except for nausea, which was statistically more frequent in the PEG-ELS group compared to the NaPico+MgCit group (*p* = 0.04) and apathy, which was statistically more frequent in the PEG-bisacodyl group compared to the NaPico+MgCit group (*p* = 0.04). All adverse events are summarized in Table 2.

### 3.3. Acceptability

Overall, all of the patients were able to complete 75% or more of the study regimen and 85.4% were able to complete the full regimen with significant difference between the PEG-ELS group compared to the NaPico+MgCit group (*p* = 0.02). The rate of children who stated that the intake of the solution was easy was significantly higher in the NaPico+MgCit group compared with both the PEG-bisacodyl group (*p* = 0.004) and the PEG-ELS group (*p* < 0.001). The acceptance of bowel cleansing agent's taste was similar in the PEG-bisacodyl group compared to the PEG-ELS group (*p* = 0.6) but was significantly lower in both PEG-based groups compared to the NaPico+MgCit group (PEG-bisacodyl vs. NaPico+MgCit: *p* = 0.007; PEG-ELS vs. NaPico+MgCit: *p* = 0.003). The rate of children who declared that the intake of the solution was difficult because of its high volume was higher in both PEG-bisacodyl group and PEG-ELS group compared to the NaPico+MgCit group (*p* = 0.03 and *p* < 0.001, respectively) and was higher in the PEG-ELS group compared to the PEG-bisacodyl group (*p* = 0.02). As a result, a significantly higher number of patients in the NaPico+MgCit group showed willingness to use the same regimen again for the next procedure compared with the PEG-ELS group (*p* < 0.001), but not with the PEG-bisacodyl group (*p* = 0.92). In PEG-based groups, the rate of children who declared that they would be willing to repeat the same preparation regimen if needed was significantly higher in the PEG-bisacodyl group compared with the PEG-ELS group (*p* < 0.001). The results of the patient acceptability questionnaire are presented in Table 3.

## 4. Discussion

The use of laxatives for adult colonoscopy is well defined, but a standardized protocol in the pediatric population is still lacking. PEG-ELS has been used for bowel cleansing in the pediatric population in Poland over the last few decades despite no approval for its use in pediatric patients in our country. Based on the earlier studies, the efficacy and safety of that medication have been confirmed [16]. However, the acceptability and tolerability of PEG-ELS which has to be mixed with a high amount of water before oral administration are limited in the pediatric population. Newer low-volume preparation alone (e.g., sodium picosulphate/magnesium citrate) or with combination with bisacodyl (e.g. PEG-bisacodyl) allows for preparation with only 2 L of solution instead of 4 L. Some of these preparations are officially approved for treatment of chronic constipation, and they have recently started to be used for bowel cleanout in children (PEG-3350) [3–12], whereas others are primarily used for colonoscopy cleansing but have no pediatric registration in Poland.

The aim of this prospective, randomized, 3-arm study was to compare the efficacy, safety, and tolerability of three different 1-day regimens of bowel preparation for the pediatric colonoscopy: high-volume PEG-ELS versus low-volume PEG-3350 without electrolytes combined with bisacodyl versus sodium picosulphate/magnesium citrate. The study represents one of the first pediatric prospective studies of sodium picosulphate/magnesium citrate for bowel cleansing. The results of the study show that high-volume PEG-ELS, low-volume PEG with bisacodyl, and sodium picosulphate/magnesium citrate have similar effectiveness of bowel preparation assessed with the BBPS score. The bowel cleansing rate of these three different regimens was at least equal to those reported in most pediatric studies [1]. Moreover, the patient tolerability of the bowel preparation assessed with the side effect rate was comparable regardless of the allocation to the group except for the significantly lower rate of nausea in children from the picosulphate/magnesium citrate group compared with the PEG-ELS group and apathy in children from the picosulphate/magnesium citrate group compared with the PEG-bisacodyl group. However, the significant difference between groups prepared with different bowel cleansing protocols was seen in relation to the acceptability. The most acceptable protocol of bowel cleansing was based on sodium picosulphate/magnesium citrate solution because of both pleasant taste and the limited volume of fluid given in a short period. In regard to the solution volume, PEG-ELS-based regimen was the worst acceptable by patients compared not only with sodium picosulphate/magnesium citrate regimen but also with PEG-bisacodyl regimen. As a result, the rate of children from the PEG-ELS group being unable to successfully complete the bowel preparation was higher than that in the sodium picosulphate/magnesium citrate group and the willingness to repeat the preparation if needed was lower when compared with the two other groups. Our results, in terms of efficacy, tolerability, and safety, are comparable with the data presented in a recently published meta-analysis on bowel preparation in children that included two studies comparing PEG and sodium picosulphate/magnesium citrate [2].

Of note, in our study, colonoscopies were performed slightly beyond 12 h after the last dose of the cleansing agent. The time interval between the end of bowel lavage and endoscopy was identified as the main factor influencing quality of bowel preparation prior to the colonoscopy. However, we do not have a possibility to administer the bowel cleansing agent as a split dose with the second half given on the morning of the endoscopy. Pediatric patients in our department undergo colonoscopy in the morning, and they are instructed by pediatric anesthesiologists to forbear from eating and drinking anything up to 4-6 hours before the procedure. Despite the long preparation-colonoscopy interval, in up to 88% of patients, a bowel cleaning success was achieved. As in our study, all of the patients were able to complete 75% or more of the recommended bowel cleansing regimen in a short period of time (up to maximum 6 hours), and the proper bowel cleansing rate was high; we suggest that the quality of bowel cleansing seems to be determined not only by the preparation-colonoscopy interval but also by the amount (%) of bowel preparation completed and the preparation drinking time. Our findings were confirmed by the outcomes of the studies with a short colonic preparation period [3, 5, 16, 17].

The present study demonstrates that all studied regimens (PEG-bisacodyl, PEG-ELS, and NaPico+MgCit) administered in a few hours are effective, tolerable, and safe. Furthermore, the efficacy of bowel cleansing in the one-day preparation regardless the type of medication was comparable to the rate reported by others for multiday protocols [4, 18–20]. The economic benefits resulting from the implementation of shorter bowel cleansing regimens and consequently the reduction of absenteeism both in schools and workplaces cannot be ignored. Therefore, the short-course bowel preparation schedules should be recommended before the pediatric colonoscopy.

It is noteworthy that despite the adverse effects reported by up to 60% of patients and suboptimal acceptance of the regimens reported by nearly 70% of patients, the percentage of children able to ingest at least 75% of the entire prescribed dose of preparations in our study was excellent. No child needed to undergo lavage via a nasogastric tube. However, the rate of pediatric patients that are unable to ingest the prescribed amount of preparation varies from study to study [4, 8, 10, 11, 14]. Several factors may explain the high compliance rate with bowel cleansing protocols in our study. Firstly, thorough verbal instructions were provided to the patient families by the physician in charge. Secondly, the bowel cleansing was overseen at the hospital by pediatric nurses. These two strategies were shown to be facilitators to an adequate bowel cleanout [21] and should be implemented to the routine clinical practice. An impact of the inpatient setting and age-related exclusion criteria on the outcomes of our study cannot be ruled out.

The incidence of adverse effects in our study was similar to the previous study results [3, 5]. As mentioned before, there were only two symptoms, e.g., nausea and apathy, which occurred significantly less frequently in the NaPico+MgCit group than in PEG-based groups with a *p* value approaching the borderline of significance. As only a few patients reported these two adverse effects in our study, the superiority of the protocol based on NaPico+MgCit over PEG-based regimens in regard to adverse effects should be interpreted with caution and needs to be confirmed in large-scale studies.

Overall, the best bowel regimen in children before the elective colonoscopy with regard to the preparation's palatability, volume of the solution, ease at taking, and willingness to repeat was the NaPico+MgCit group. The results are in line with previous studies confirming that bowel cleansing with picosulphate/magnesium citrate was better tolerated and accepted than protocols with polyethylene glycol among children between 10 and 18 years of age [2, 9, 12–14].

Our study has strengths and limitations. The strength of the study is the utilisation of the Boston Bowel Preparation Score for the assessment of cleansing efficacy. The scale was previously validated in adults. It applies metrics of the colonic cleanliness during the withdrawal of the endoscope after suctioning and washing the bowel mucosa, thus retained fecal material and liquid do not affect the bowel cleansing score. It also demonstrated good intra- and interobserver reliability among physicians [15]. No additional stimulant, enemas, or suppositories potentially influencing the results were administered to the pediatric patients in the present study. The assessment of the efficacy was performed by two endoscopists blinded for the preparation method; thus, it was less susceptible to the performance bias.

The small sample size is an important limitation of our study. Future studies with a larger sample size are necessary to confirm the results and are planned to be conducted. Moreover, the study did not assess safety by measuring laboratory parameters. However, the preparations used in our study are industrially supplemented by electrolytes (PEG-ELS, sodium picosulphate/magnesium citrate) or were administered with electrolyte-containing liquids. As it was pointed out, sports drinks have fewer electrolytes, increased carbohydrates, and flavorings that may alter osmolarity [22]; therefore, in the PEG-bisacodyl group, it was decided to mix PEG-3350 with weight-adjusted isotonic electrolyte-containing fluid used for intravenous administration instead of sports drinks. Therefore, no clinically significant laboratory changes after bowel preparation were expected. Other authors also did not find clinically significant electrolyte shifts in their population, although patients with risk factors for complications (e.g., younger children, children with kidney disease) were excluded [3–7, 9]. The only laboratory disturbance reported by some authors [6] was hypoglycemia detected in children of 7 years of age and younger prepared for the elective colonoscopy with PEG-3350 without added electrolyte solution. However, in our study, the risk related to hypoglycemia was avoided by glucose-containing intravenous fluid administration under general anesthesia.

The other limitation of the study is the mean age of the study participants (14.1). The group of children below the age of 10 was excluded from the study. However, this is the most challenging and potentially vulnerable group of patients with risk factors for complications and inadequate preparation, and thus, it is believed to require a distinct bowel cleansing regimen. Moreover, all of the patients were enrolled from a single, tertiary medical care center, and the overwhelming majority of patients were referred for elective colonoscopy with suspected inflammatory bowel disease with no previous history of constipation, and thus, the outcomes of our study group may not be generalized to the entire pediatric population.

In this randomized, prospective study, we found that high-volume PEG, low-volume-PEG, and NaPico were efficient in terms of bowel cleanout in children between 10 and 18 years of age, although the use of NaPico resulted in better acceptance and tolerability than PEG-based regimens. Future studies are warranted to evaluate whether NaPico can be recommended as a colonic cleansing agent for children below 10 years of age. There is still a need for careful evaluation of new and existing protocols to provide tolerable, convenient, and effective preparation with minimal adverse events.

## Figures and Tables

**Table 1:** Baseline characteristics of study groups.

Parameter	PEG-bisacodyl group	PEG-ELS group	NaPico+MgCit group	*p*
*N*	41	43	39	
Mean age (SD) (years)	13.9 (2.4)	14.5 (2.2)	13.9 (2.7)	0.4
Min–Max	10-17	10-17	10-17	
Sex, males (%)	24 (58.5)	22 (51.2)	16 (41.0)	0.1
Weight: mean (kg)	51.6	56.2	51.7	0.3
Reason for colonoscopy				
IBD: *N* (%)	34 (82.9)	36 (83.7)	33 (84.6)	0.9
Other: *N* (%)	7 (17.1)	7 (16.3)	6 (15.4)	

**Table 2:** Safety of three bowel cleansing regimens.

Adverse event	PEG-bisacodyl group, *N* = 41	PEG-ELS group, *N* = 43	NaPico+MgCit group, *N* = 39	All groups, *N* = 123	*p*
Overall	25 (61%)	30 (69.8%)	18 (46.2%)	73 (59.3%)	^∗^
Abdominal pain	13 (31.7%)	16 (37.2%)	13 (33.3%)	42 (34.1%)	NS
Nausea	7 (17.1%)	8 (18.6%)	0 (0%)	15 (12.2%)	^∗∗^
Vomiting	6 (14.6%)	4 (9.3%)	2 (5.1%)	12 (9.8%)	NS
Flatulence	4 (9.8%)	4 (9.3%)	0 (0%)	8 (6.5%)	NS
Perianal discomfort/pain	8 (19.5%)	3 (7%)	4 (10.3%)	15 (12.2%)	NS
Sleep disturbance	14 (34.1%)	16 (37.2%)	16 (37.2%)	7 (17.9%)	NS
Dizziness	7 (17.1%)	3 (7.0%)	1 (2.6%)	11 (8.9%)	NS
Apathy	9 (22.2%)	5 (11.6%)	2 (5.1%)	16 (13.0%)	^∗∗∗^
Others	2 (4.9%)	2 (4.7%)	2 (5.1%)	6 (4.9%)	NS

NS = statistically nonsignificant; ^∗^PEG-bisacodyl vs. PEG-ELS: *p* = 0.4, PEG-bisacodyl vs. NaPico+MgCit: *p* = 0.2, and PEG-ELS vs. NaPico+MgCit: *p* = 0.03; ^∗∗^PEG-bisacodyl vs. PEG-ELS: *p* = 0.9, PEG-bisacodyl vs. NaPico+MgCit: *p* = 0.06, and PEG-ELS vs. NaPico+MgCit: *p* = 0.04; ^∗∗∗^PEG-bisacodyl vs. PEG-ELS: *p* = 0.2, PEG-bisacodyl vs. NaPico+MgCit: *p* = 0.04, and PEG-ELS vs. NaPico+MgCit: *p* = 0.3.

**Table 3:** Patient acceptability questionnaire.

Variable	PEG-bisacodyl group, *N* = 41	PEG-ELS group, *N* = 43	NaPico+MgCit group, *N* = 39	All groups, *N* = 123	*p*
Ease of taking					
No distress	11 (26.8%)	7 (16.3%)	23 (59.0%)	41 (33.3%)	^∗^
Mild difficulty	9 (23%)	14 (32.6%)	8 (20.5%)	31 (25.2%)	NS
Moderate difficulty	10 (24.4%)	11 (25.6%)	5 (12.8%)	26 (21.1%)	NS
Great difficulty	11 (26.8%)	11 (25.6%)	4 (10.3%)	26 (21.1)	NS
Poor acceptance of taste	13 (31.7%)	16 (37.2%)	2 (5.1%)	31 (25.2%)	^∗∗^
High volume	10 (24.4%)	21 (56.8%)	2 (5.1%)	33 (28.2%)	^∗∗∗^
Whole volume intake	36 (87.8%)	32 (74.4%)	37 (94.9%)	105 (85.4%)	^±^
Willingness to repeat	25 (61.0%)	16 (37.2%)	33 (84.6%)	74 (60.2%)	^±±^

NS = statistically nonsignificant; ^∗^PEG-bisacodyl vs. PEG-ELS: *p* = 0.2, PEG-bisacodyl vs. NaPico+MgCit: *p* = 0.004, and PEG-ELS vs. NaPico+MgCit: *p* < 0.001; ^∗∗^PEG-bisacodyl vs. PEG-ELS: *p* = 0.6, PEG-bisacodyl vs. NaPico+MgCit: *p* = 0.007, and PEG-ELS vs. NaPico+MgCit: *p* = 0.003; ^∗∗∗^PEG-bisacodyl vs. PEG-ELS: *p* = 0.02, PEG-bisacodyl vs. NaPico+MgCit: *p* = 0.03, and PEG-ELS vs. NaPico+MgCit: *p* < 0.001; ^±^PEG-bisacodyl vs. PEG-ELS: *p* = 0.1, PEG-bisacodyl vs. NaPico+MgCit: *p* = 0.3, and PEG-ELS vs. NaPico+MgCit: *p* = 0.02; ^±±^PEG-bisacodyl vs. PEG-ELS: *p* < 0.001, PEG-bisacodyl vs. NaPico+MgCit: *p* = 0.9, and PEG-ELS vs. NaPico+MgCit: *p* < 0.001.

## Data Availability

The data used to support the findings of this study are available from the corresponding author upon request.
