# Corrigendum to “The Effectiveness, Tolerability, and Safety of Different 1-Day Bowel Preparation Regimens for Pediatric Colonoscopy”

**DOI:** 10.1155/2020/9126957

**Published:** 2020-06-18

**Authors:** Anna Szaflarska-Poplawska, Dominika Tunowska, Ola Sobieska-Poszwa, Anna Gorecka, Aneta Krogulska

**Affiliations:** ^1^Department of Pediatric Endoscopy and Gastrointestinal Function Testing, Ludwik Rydygier Collegium Medicum in Bydgoszcz, Nicolaus Copernicus University in Torun, Poland; ^2^Department of Pediatrics, Allergology and Gastroenterology, Ludwik Rydygier Collegium Medicum in Bydgoszcz, Nicolaus Copernicus University in Torun, Poland

In the article titled “The Effectiveness, Tolerability, and Safety of Different 1-Day Bowel Preparation Regimens for Pediatric Colonoscopy” [1], Anna Gorecka was missing from the author list. The corrected author list is shown above.
